# Restored intestinal integrity, nutrients transporters, energy metabolism, antioxidative capacity and decreased harmful microbiota were associated with IUGR piglet's catch-up growth before weanling

**DOI:** 10.1186/s40104-022-00770-8

**Published:** 2022-10-14

**Authors:** Chang Cui, Caichi Wu, Jun Wang, Ziwei Ma, Xiaoyu Zheng, Pengwei Zhu, Nuan Wang, Yuhua Zhu, Wutai Guan, Fang Chen

**Affiliations:** 1 Guangdong Provincial Key Laboratory of Animal Nutrition Control, College of Animal Science, South China Agricultural University, Guangzhou, 510642 China; 2 Shenzhen Kingsino Technology CO., LTD, Shenzhen, 518107 China; 3 Shenzhen Institute of Nutrition and Health, Huazhong Agricultural University, Wuhan, 430070 China; 4 Agricultural Genomics Institute at Shenzhen, Chinese Academy of Agricultural Sciences, Shenzhen, 518116 China; 5 College of Animal Science and National Engineering Research Center for Breeding Swine Industry, South China Agricultural University, Guangzhou, 510642 China; 6 Guangdong Laboratory for Lingnan Modern Agriculture, South China Agricultural University, Guangzhou, 510642 China

**Keywords:** Antioxidative capacity, Catch-up growth, Gut, Intrauterine growth retardation, Piglets

## Abstract

**Background:**

Intrauterine growth restriction (IUGR) is a major inducer of higher morbidity and mortality in the pig industry and catch-up growth (CUG) before weanling could significantly restore this negative influence. But there was limited knowledge about the underlying mechanism of CUG occurrence.

**Methods:**

Eighty litters of newborn piglets were divided into normal birth weight (NBW) and IUGR groups according to birth weight. At 26 d, those piglets with IUGR but over average body weight of eighty litters of weaned piglets were considered as CUG, and the piglets with IUGR still below average body weight were considered as NCUG. This study was conducted to systemically compare the intestinal difference among NBW, CUG and NCUG weaned piglets considering the crucial role of the intestine for piglet growth.

**Results:**

The results indicated that the mRNA expression of nutrients (amino acids, glucose, and fatty acids) transporters, and mitochondrial electron transport chain (ETC) I were upregulated in CUG piglets’ gut with improved morphology compared with those NCUG, as well as the ratio of P-AMPK/AMPK protein expression which is the indicator of energy metabolism. Meanwhile, CUG piglet’s gut showed higher antioxidative capacity with increased SOD and GSH-Px activity, decreased MDA levels, as well as higher mRNA expressions of *Nrf2*, *Keap1*, *SOD,* and *GSH-Px*. Furthermore, inflammatory parameters including *TNF-α*, *IL-1β*, *IL-6,* and *IL-12* factors, and the activation of MAPK and NF-κB signaling pathways were significantly elevated in the NCUG intestine, while the protein expression of ZO-1, Occludin and Claudin-1 was reduced. The alpha diversity of fecal microbiota was higher in CUG piglets in contrast with NCUG piglets, and the increased beneficial bacteria and decreased pathogenic bacteria was also observed in CUG piglets.

**Conclusions:**

CUG piglet’s intestine showed comprehensive restoration including higher nutrients transport, energy metabolism, antioxidant capacity, and intestinal physical barrier, while lower oxidative stress, inflammatory response, and pathogenic microbiota.

## Background

Intrauterine growth restriction (IUGR) is defined as the limited growth and development of mammalian fetus and organs during pregnancy and born with smaller body weight [1]. It has been well established that IUGR fetus are usually accompanied by comprehensive negative postnatal outcomes and metabolic disorders in various species [1–3]. As multiparous animals, pigs are most prone to IUGR and its occurrence has been reported up to 15%–20%, which might continue to raise due to the bigger litter size attributed to the improvement of breeding science nowadays [1]. The weaning morbidity of piglets with IUGR was about 11%, which was significantly higher than that of piglets without IUGR, causing huge economic loss to animal husbandry [1, 4]. Catch-up growth (CUG) refers to the body’s rapid growth after a period of growth inhibition, which has been observed in a portion of IUGR newborns in their early postnatal life [5, 6]. Previous studies have shown that CUG infants are 65% less likely to be hospitalized in the first 20 months of birth and 75% less likely to die at age 6 than those infants still with smaller body weight at that time, which was a considerable benefit for human health [7]. Limited results in pigs also showed that early postnatal CUG has similar morbidity and growth potential as normal birth weight (NBW) piglets at birth during growth and fattening period [8], suggesting that the importance of CUG in pigs should be addressed especially before weanling to reduce economic loss caused by IUGR in swine production.

Gut plays a crucial role in nutrient digestion and absorption directly contributing to piglet growth, and its abnormal development is closely associated with high diarrhea and mortality rates in weaned piglets, as well as impaired overall health and productive performance [9]. It has been reported that newborn IUGR pigs have significantly lower intestinal weight compared with that of NBW piglets [10], and this stunned intestinal development subsequently continued during the whole later life in the absence of CUG [11–13]. Besides, IUGR has shown negative effects on the expression of proteins involved in the absorption, digestion, and transportation of nutrients and is continuously impaired during lactation [14, 15]. To date, a large number of studies explored the mechanism of impaired intestinal development in IUGR pigs from abroad aspects, including immune and inflammatory system, cellular apoptosis signal transduction, protein synthesis, as well as microbial diversity [1, 15, 16], which provide great help to understand the difference between NBW and IUGR piglets and better take care of IUGR piglets to reduce their mortality and morbidity. However, to our knowledge, there were very limited investigations focusing on the underlying mechanisms about CUG of piglets, which is of importance and significance for pig industry to explore new strategies to improve productive performance by increasing the occurrence rate of this beneficial phenomenon.

Hence, we used different growth patterns IUGR (CUG and NCUG before weaning) and NBW piglets as animal models in the present study to systematically compare the intestinal difference in morphology, barrier function, antioxidant status, inflammation levels, nutrient transport, energy metabolism, and fecal microbiome. Moreover, considering that most previous studies mainly focused on duodenum and jejunum function in IUGR piglets [2, 17, 18], but paid little attention in the ileum. The ileum has recently been reported closely related to viral intestinal diseases and has attracted more and more attention. Therefore, three segments of intestine including duodenum, jejunum and ileum were all collected in current study for analysis due to their different physiological function. This study may also shed light on the detailed understanding of gut development and adaption for CUG infants.

## Materials and methods

### Animals and experimental design

**Fig. 1:**
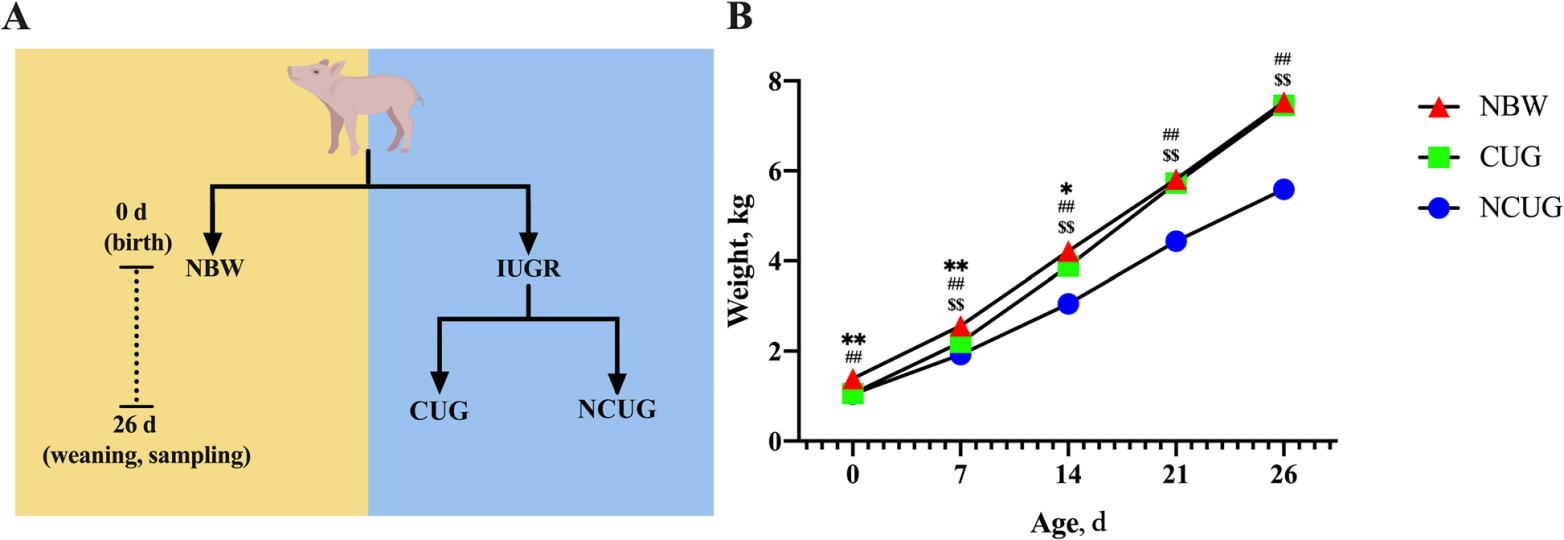
Experiment design and the establishment of CUG model. **A** Schematic representation of the experimental design. **B** Body weight from birth to postnatal 26 d of NBW, CUG and NCUG piglets. NBW, normal birth weight. IUGR, intrauterine growth retardation. CUG, catch-up growth. NCUG, not catch-up growth. *P* < 0.05, ^*^NBW vs. CUG-IUGR. *P* < 0.01, ^**^NBW vs. CUG, ^##^NBW vs. NCUG, ^$^^$^CUG vs. NCUG

All animal procedures were carried out in accordance with the Guidelines for Care and Use of Laboratory Animals of South China Agricultural University and approved by the Animal Ethics Committee of South China Agricultural University (No. 20110107–1, Guangzhou, China). During gestation, 80 healthy pregnant sows (Landrace × Yorkshire) were preselected according to similar due dates and parity (2–3). The newborn piglets were weighed immediately after delivery. IUGR piglets were defined as the average birth weight was 2 standard deviations below the average birth weight of the total population, and NBW piglets are defined as the birth within 0.5 standard deviations of the average birth weight of the total population. According to the birth weight, 55 IUGR piglets and 28 NBW piglets weighing more than 1.2 kg were selected to participate in this study. 55 IUGR piglets were separated from NBW piglets and fostered to 6 sows with similar body conditions during lactation. All the piglets were weighed and measured weekly to track their growth pattern. On the weaning day (26 d), 14 IUGR piglets with weaning weight exceeding the average body weight of 80 litters of weaned piglets were divided into the CUG group, and 33 IUGR piglets with weaning weight not exceeding the average body weight of 80 litters of weaned piglets were divided into the NCUG group (Fig. 1A).

### Sample collection

At age of 7 days, all IUGR piglets were included in this study to collect feces by separating them one by one into a clean crate, then administered rectal stimulation with a sterile swab, and collected feces at least 5 g directly into a sterile centrifuge tube. After weaning, 3 weaned piglets were randomly selected from CUG and NCUG piglets to perform 16S rRNA sequencing analysis for their feces samples collected at the age of 7 days. At age of 26 days, 6 weaned piglets were randomly selected from each group of NBW, CUG, and NCUG groups for the slaughter experiment. After intravenous injection of 100 mg/kg lethal dose of sodium pentobarbital, the abdominal cavity was opened. The duodenum was considered as 10 cm away from the gastric pylorus, the jejunum was considered as 40 cm away from the cecum, and the ileum was considered as 10 cm away from the cecum [19]. About 1 cm long samples were collected at the middle part of the duodenum, jejunum, and ileum and then fast stored in 4% paraformaldehyde fix solution for subsequent morphological observation. The active component of 4% paraformaldehyde, using 0.1 mol/L phosphoric acid buffer as solvent, pH 7.0–7.5 at 25 °C. At the same time, each intestinal segment about 2 cm was opened longitudinally, and the intestinal contents were rinsed three times using cold normal saline, then quickly frozen in liquid nitrogen for mRNA and protein expression analysis. The time from euthanasia to complete sampling was controlled at about 30 min for each piglet.

### Intestinal morphology

Duodenum, jejunum, and ileum samples were stored in 4% paraformaldehyde fix solution for morphological analysis. Standard paraffin embedding procedures and standard hematoxylin and eosin staining protocols were used. In simple terms, intestinal tissues were dehydrated in a series of ethanol diluents, washed with xylene, and then embedded in paraffin wax. Paraffin samples were cut into 5 μm sections and stained with hematoxylin and eosin. Two discontinuous sections were selected from each tissue, and 5 representative villi and their associated crypt were selected from each section. Villi height (VH) and crypt depth (CD) were viewed on the light microscope and measured using an image processing and analysis system (NIS-Elements Viewer, Tokyo, Japan).

### Real time quantitative PCR

The total RNA was isolated from duodenum, jejunum and ileum using the Tissue RNA Purification kit PLUS (EZB-RN001-plus, EZBioscience, Roseville, MN, USA) followed the manufacturer’s instruction. The extracted RNA quality and concentration were evaluated with 1.0% agarose gel electrophoresis (130 V, 18 min), and the absorption ratio of RNA (A_260_/A_280_) for all RNA samples was greater than 1.8 and less than 2.3 by using a spectrophotometer. The cDNA synthesis was performed by the RNA reverse transcription reaction with the Color Reverse Transcription Kit (A0010CGQ, EZBioscience, Roseville, MN, USA), according to the manufacturer’s protocol. Real time PCR was conducted using ABI Prism 7500 sequence detection system (Applied Biosystems, Carlsbad, CA, USA) with a reaction volume of 20 μL. The PCR reaction scheme includes initial denaturation one cycle at 95 °C for 2 min, amplification forty cycles at 95 °C for 15 s, 60 °C for 30 s. The relative target gene expression levels were determined based on the quantification approach (2^−ΔΔCt^ method), with *β-actin* acting as the housekeeping gene to normalize all mRNA levels. The total primers used are presented in Table 1 [20–22].

**Table 1:** Primer sequences used in Real-time PCR

Genes	Accession	Forward primer(5’ → 3’)	Reverse primer (5’ → 3’)
*LAT1*	NM_003486	GCCCATTGTCACCATCATC	GAGCCCACAAAGAAAAGC
*CAT1*	NW_003611328.1	GCCTGAGAGCAAGACCAAAC	GCCGTAGCCGAAGTAGATGA
*EAAC1*	NM_001164649.1	GTTCCTGATTGCCGGGAAGA	ATGGCGAATCGGAAAGGGTT
*PepT1*	AY180903.1	AGCATCTTCTTCATCGTG GTCAA	GTCTTGAACTTCCCCAGCCA
*SGLT1*	NM_001164021	CATCATCGTCCTGGTCGTC	CATCATCGTCCTGGTCGTC
*GLUT2*	EF140874	GTCCAGAAAGCCCAAGAT ACC	GTGACATCATCACTTCCTCTGAG
*CD36*	NM_001083931.1	GGCAACAGACGTGATCTATGAC	AGCGGCTGGCTGAAAACT
*FATP4*	XM_003353676.1	AGCCGCATCCTGTCCTTT	GACATCCTTGGCGATCTTTT
*NDUF A1*	XM_003135339.4	GCTTCCGGGGAAGGAATCAA	CCGGGGAGAATTTCGAACCA
*NDUF A6*	NM_001185178.1	TCTCAGAGCCTTGCATGTCG	AAGCCATCCAGCATCGTACC
*NDUF A13*	NM_001244646.1	ATGAAGGATGTGCCGGACTG	CCATAGGTGGCGCTGAGAAT
*NDUF B1*	XM_003482306.3	TGCCTTCCGGAACAAGAGTC	GCAATTCAGCCACAGCCTTT
*Nrf2*	NM_001114671.1	GACAAACCGCCTCAACTCAG	GTCTCCACGTCGTAGCGTTC
*Keap1*	XM_021076667.1	CGTGGAGACAGAAACGTGGA	CAATCTGCTTCCGACAGGGT
*SOD*	NM_001190422.1	AAGGCCGTGTGTGTGCTGAA	GATCACCTTCAGCCAGTCCTTT
*GSH-Px*	NM_214201.1	CCTCAAGTACGTCCGACCAG	GTGAGCATTTGCGCCATTCA
*TNF-α*	NM_214022.1	CCACCAACGTTTTCCTCACT	TAGTCGGGCAGGTTGATCTC
*IL-1β*	NM_214055.1	CCAAAGAGGGACATGGAGAA	TTATATCTTGGCGGCCTTTG
*IL-6*	NM_001252429.1	TGGCTACTGCCTTCCCTACC	AGAGCCTGCATCAGCTCAGT
*IL-12*	NC_010458.4	CAACCCTGTGCCTTAGCAGT	AGAGCCTGCATCAGCTCAGT
*MAPK3*	XM_021088019.1	CAGTCTCTGCCCTCCAAGAC	GGGTAGATCATCCAGCTCCA
*MAPK8*	XM_001929166.6	TGGATGAAAGGGAACACACA	ATGATGACGATGGATGCTGA
*MAPK14*	XM_021091323.1	CCCTGAGGTTCTAGCGAAGA	TCTCATCGTAGGGCTCTGCT
*NFKB1*	NM_001048232.1	CTCGCACAAGGAGACATGAA	ACTCAGCCGGAAGGCATTAT
*β-actin*	XM_021086047.1	TGCGGGACATCAAGGAGAAG	AGTTGAAGGTGGTCTCGTG

### Western blot

Total protein was extracted from the duodenal, jejunal and ileal tissues and homogenized by adding a mixture of RIPA lysis buffer (P0013B, Beyotime, Shanghai, China) containing protease inhibitor PMSF (ST506, Beyotime, Shanghai, China). The protein concentration in supernatant was measured using a BCA Protein Assay Kit (P0010, Beyotime, Shanghai, China) after the centrifuge. Thereafter, the equal quantities of protein (25 μg) were separated by 10% SDS-PAGE gels and electrically transferred onto polyvinylidene difluoride (PVDF) membranes. After that, the membranes were blocked with 5% skimmed milk for 2 h at room temperature and then incubated with the primary antibody against Claudin-1 (1:1000, ab129119, Abcam, Cambridge, UK), ZO-1(1:1000, 21773–1-AP, Proteintech, Wuhan, China), Occludin (1:1000, 27260–1-AP, Proteintech, Wuhan, China), P-JNK (1:1000, 4688S, Cell Signaling Technology, Boston, MA, USA), JNK (1:1000, 9252S, Cell Signaling Technology, Boston, MA, USA), P-NF-κB (1:1000, 3033S, Cell Signaling Technology, Boston, MA, USA), NF-κB (1:1000, 10745–1-AP, Proteintech, Wuhan, China), P-AMPK(1:1000, 2535S, Cell Signaling Technology, Boston, MA, USA), AMPK (1:1000, 2432S, Cell Signaling Technology, Boston, MA, USA) and β-actin (1:2000, bs-0061R, Bioss, Beijing, China) overnight at 4 °C. Then, the membranes were washed by Tris buffered saline Tween and incubated with a corresponding secondary antibody (1:50,000, 511203, ZenBio, Chengdu, China) for 1.5 h at room temperature, followed by visualizing the target bands using an enhanced chemiluminescence kit (P1020, Applygen, Beijing, China) using the ImageQuant LAS 4000 mini system.

### Antioxidant status

The duodenal, jejunal and ileal tissues were homogenized with saline solution (1:4, weight:volume) and centrifuged at 3000 × *g* for 15 min. According to the kit instructions, the supernatants were diluted to the optimal concentration for detecting the activities of glutathione peroxidase (GSH-PX), reduced glutathione (GSH), superoxide dismutase (SOD), total antioxidant capacity (T-AOC) and malonaldehyde (MDA) by commercially available kits (Nanjing Jiancheng Bioengineering Institute, Nanjing, China).

### 16S rRNA Sequencing

Total genomic DNA was extracted from samples by CTAB method [23]. The concentration and purity of DNA were monitored on 1% agarose gels. V3-V4 variable regions of 16S rRNA genes were amplified with primers 341F (5’-CCTAYGGGRBGCASCAG-3’) and 806R (5’- GGACTACNNGGGTATCTAAT-3’). 15 μL Phusion® High-Fidelity PCR Master Mix (New England Biolabs, Beijing, China) was used for PCR reaction. The primers of 341F and 806R mentioned above were 2 μmol/L, and the template DNA was about 10 ng. The thermal cycles include initial denaturation at 98 °C for 1 min, denaturation at 98 °C for 10 s, annealing at 50 °C for 30 s, and extension at 72 °C for 30 s. Finally, 72 °C for 5 min. An equal amount of 1× loading buffer was mixed with PCR products and electrophoresis was performed on 2% agarose gel. PCR products were mixed at an isodensity ratio. The mixed PCR products were purified using the Qiagen Gel Extraction Kit (Qiagen, Dusseldorf, Germany). Sequencing libraries were generated by TruSeq® DNA PCR-Free Sample Preparation Kit (Illumina, San Diego, California, USA) according to manufacturer's recommendations and index codes were added. The library quality was evaluated on the Qubit@ 2.0 Fluorometer (Thermo Scientific, Waltham, MA, USA) and Agilent Bioanalyzer 2100 system. Finally, the library was sequenced on an Illumina Nova Seq platform to obtain 250 bp paired-end reads. Paired-end reads were performed on samples based on their unique barcodes and truncated by truncating barcodes and primer sequences. Paired-end reads were spliced using the FLASH software to obtain Raw Tags. Date filtration and noise reduction were performed on DADA2 module of QIME software (Version 1.9.1), then the ASVs and their feature table are obtained. The obtained ASVs were annotated for species, and finally the species information of each ASV was obtained.

### Statistical analysis

All data in the experiment except microbial part were analyzed by one-way ANOVA using SPSS 22.0 (IBM Inc., Armonk, New York, USA) to determine whether significant differences among the groups. The data were expressed by means ± SEM. Correlations were evaluated by Pearson correlation analysis of the Euclidean distance using GraphPad Prism 9.0 (GraphPad Software, San Diego, CA, USA). *P* < 0.05 was considered a significant difference and highly significant when *P* < 0.01.

Alpha diversity, including Chao1, Shannon, and Simpson was used to analyze the complexity of species diversity. All alpha diversity indices in our samples were calculated with QIIME (Version 1.7.0) and displayed with R software (Version 2.15.3). Beta diversity analysis was used to assess differences of samples in species complexity, and beta diversity on weighted_unifrac was performed using QIIME software (Version 1.9.1). Principal coordinate analysis (PCoA) was performed to get principal coordinates and visualize from complex, multidimensional data. *T* test statistical algorithm was used to analyze differences. Linear discriminant analysis (LDA) was used to identify the bacterial groups in each group by LEfSe.

## Results

### Establishment of piglet models with different growth patterns

The birth weight of IUGR piglets including CUG and NCUG piglets was significantly lower than that NBW piglets (*P* < 0.01), and there was no difference between CUG piglets and NBW piglets at the weanling day (26 d), indicating that CUG and NCUG models were successfully established (Fig. 1B).

### Intestinal morphology

**Fig. 2:**
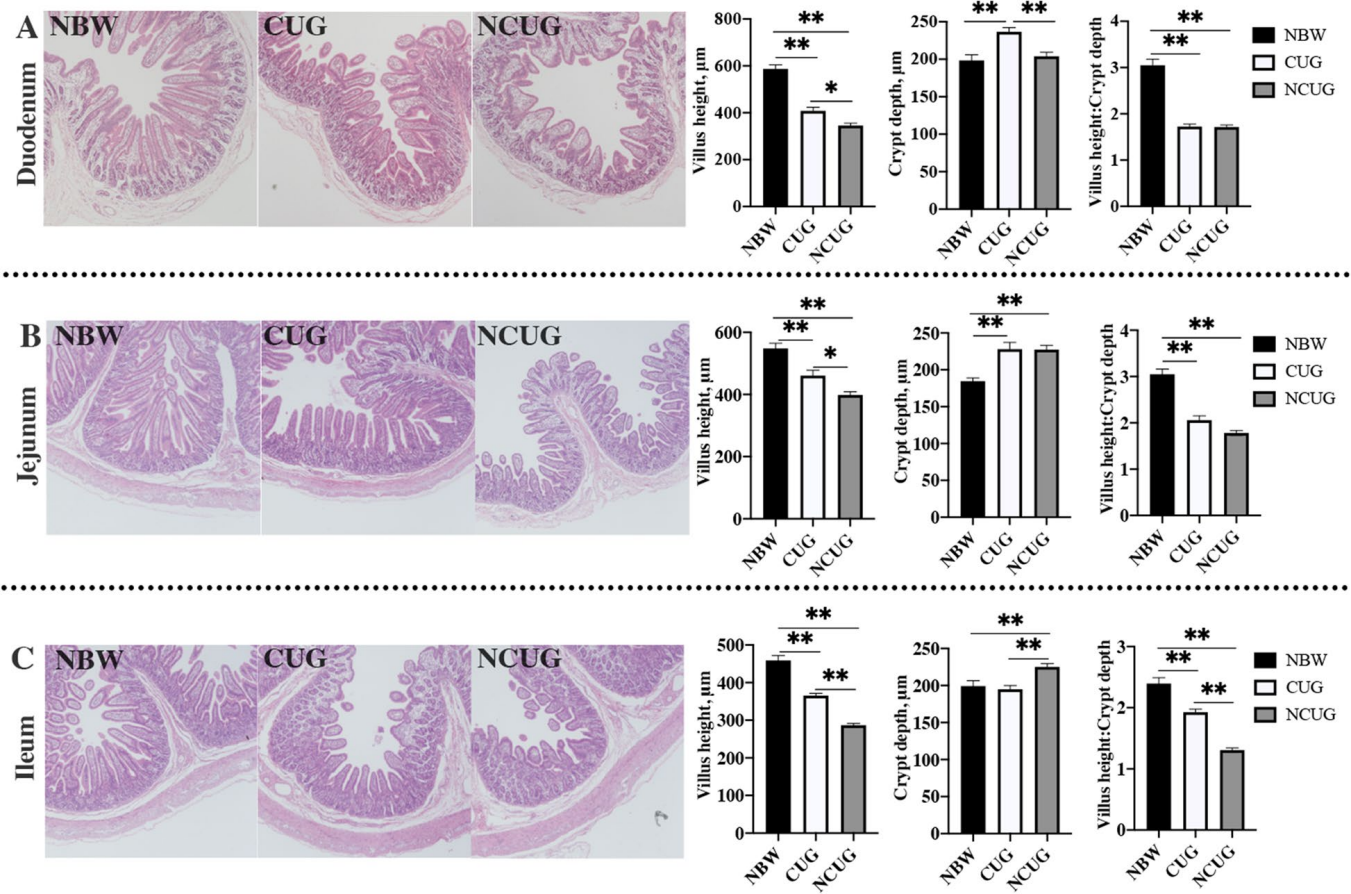
Intestinal morphology of NBW, CUG and NCUG piglets on 26 d. **A** Duodenal morphology, villus height, crypt depth, ratio of villus height to crypt depth. **B** Jejunal morphology, villus height, crypt depth, ratio of villus height to crypt depth. **C** Ileal morphology, villus height, crypt depth, ratio of villus height to crypt depth. Dates are presented as means ± SEM (*n* = 6). NBW, normal birth weight. CUG, catch-up growth, NCUG, not catch-up growth. *P < 0.05; **P < 0.01

Obvious decreases in VH and VH/CD ratio in duodenum, jejunum, and ileum of CUG and NCUG piglets were observed compared with the NBW (Fig. 2; *P* < 0.05). In contrast with NBW piglets, the CD was significantly increased in duodenum of CUG piglets, jejunum of CUG and NCUG piglets, and ileum of NCUG piglets (*P* < 0.05). Simultaneously, higher VH in the duodenum, jejunum and ileum was observed, as well as lower CD in ileum, but higher CD in duodenum and VH/CD ratio in ileum of CUG piglets compared with NCUG counterparts (*P* < 0.05). The VH/CD ratio in the duodenum and jejunum were similar between the CUG and NCUG groups (*P* > 0.05).

### Intestinal nutrients transport mRNA expression

**Fig. 3:**
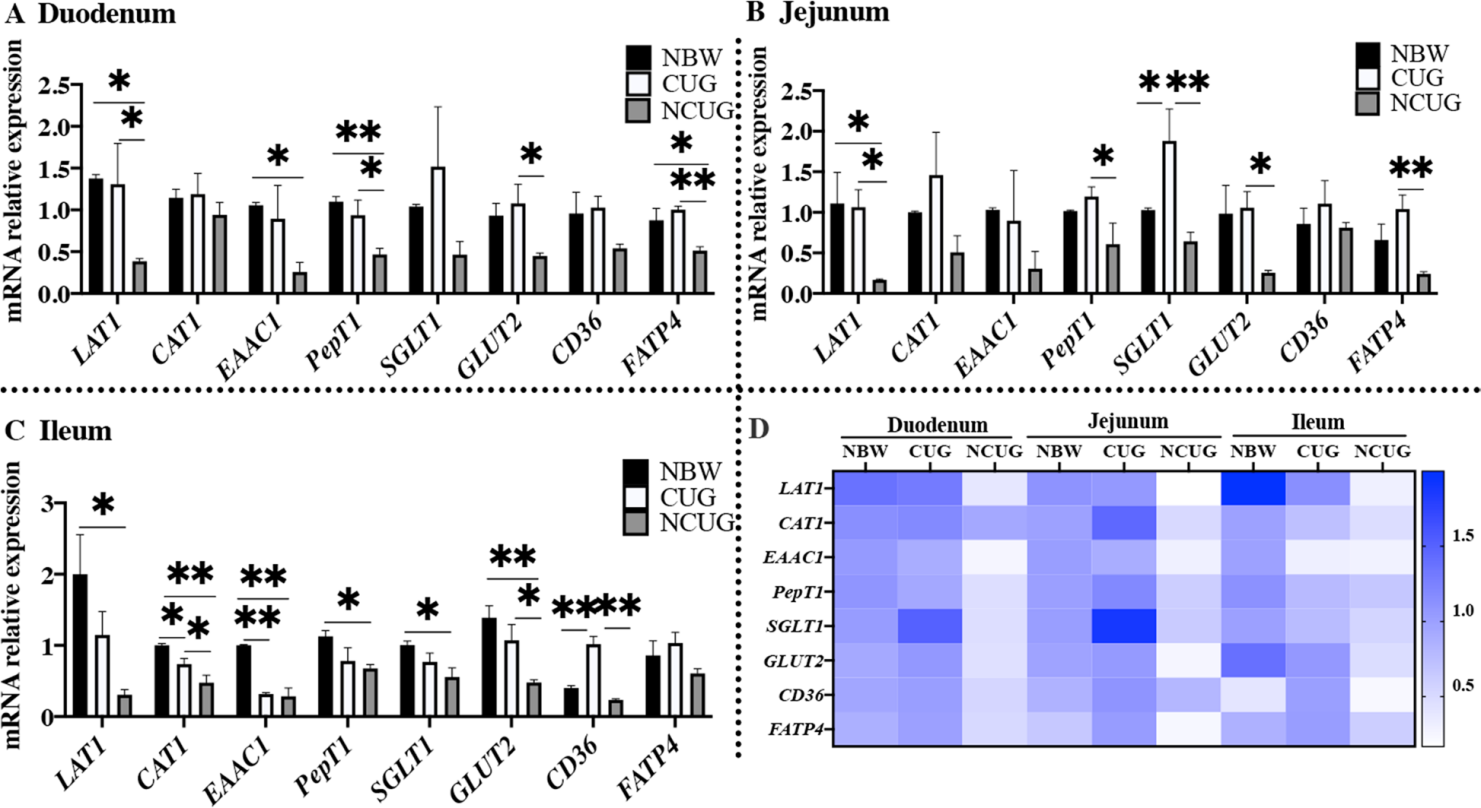
Intestinal nutrition transporters mRNA expression of NBW, CUG and NCUG piglets on 26 d. **A** The mRNA expression levels of *LAT1*, *CAT1*, *EAAC1*, *PepT1*, *SGLT1*, *GLUT2*, *CD36* and *FATP4* in the duodenum. **B** The mRNA expression levels of *LAT1*, *CAT1*, *EAAC1*, *PepT1*, *SGLT1*, *GLUT2*, *CD36* and *FATP4* in the jejunum. **C** The mRNA expression levels of *LAT1*, *CAT1*, *EAAC1*, *PepT1*, *SGLT1*, *GLUT2*, *CD36* and *FATP4* in the ileum. **D** Heat map comparison of mRNA expression levels of nutrition transporters in NBW, CUG and NCUG. Dates are presented as means ± SEM (*n* = 4). NBW, normal birth weight. CUG, catch-up growth. NCUG, not catch-up growth. *P < 0.05; **P < 0.01

Gut plays a crucial role in nutrient transport, which processes are mainly regulated by specific amino acids transporters, glucose transporters and fatty acids transporters located in intestine. At weaning (26 d), compared to NBW group, piglets with NCUG had a significantly lower expression of duodenal *LAT1*, *EAAC1*, *PepT1* and *FATP4*, jejunal *LAT1*, ileal *LAT1*, *CAT1*, *EAAC1*, *PepT1*, *SGLT1* and *GLUT2* (Fig. 3A, B, C; *P* < 0.05). CUG piglets had significantly higher expression of *SGLT1* in the jejunum, significantly lower expression of *CAT1*, *EAAC1* in the ileum, and significantly higher expression of *CD36* in the ileum compared with NBW piglets (*P* < 0.05). Specifically, CUG group showed higher mRNA expression levels of duodenal and jejunal *LAT1*, *PepT1*, *GLUT2* and *FATP*, ileal *CAT1*, *GLUT2* and *CD36* in contrast with the NCUG group (*P* < 0.05).

### The mRNA and proteins associated with energy metabolism

**Fig. 4:**
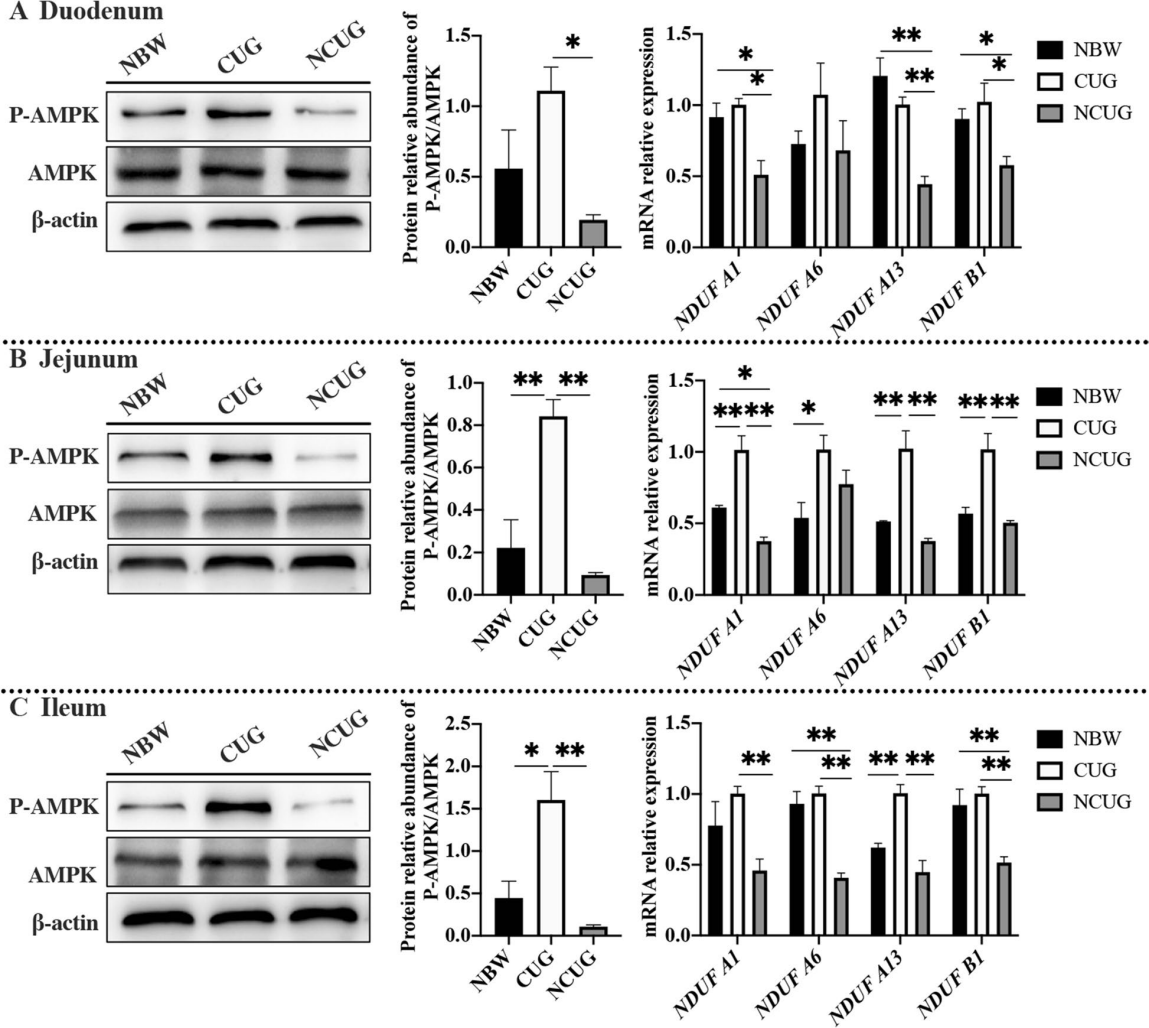
The intestinal mRNA and proteins expression associated with energy metabolism of NBW, CUG and NCUG piglets on 26 d. **A** Duodenal protein expression of P-AMPK/AMPK and mRNA expression of mitochondrial electron transport chain I, including *NDUF A1*, *NDUF A6*, *NDUF A13* and *NDUF B1*. **B** Jejunal protein expression of P-AMPK/AMPK and mRNA expression of mitochondrial electron transport chain I, including *NDUF A1*, *NDUF A6*, *NDUF A13* and *NDUF B1*. **C** Ileal protein expression of P-AMPK/AMPK and mRNA expression of mitochondrial electron transport chain I, including *NDUF A1*, *NDUF A6*, *NDUF A13* and *NDUF B1*. Dates are presented as means ± SEM (*n* = 3 for protein expression; *n* = 4 for mRNA expression). NBW, normal birth weight. CUG, catch-up growth. NCUG, not catch-up growth. *P < 0.05; **P < 0.01

To detect the function of mitochondria and AMPK in energy metabolism, the mRNA expression of mitochondrial electron transport chain (ETC) I and the protein expression of P-AMPK/AMPK were detected. The data indicated lower mRNA expression levels of *NDUF A1*, *NDUF A13* and *NDUF B1* in the duodenum, *NDUF A1* in the jejunum, *NDUF A6,* and *NDUF B1* in the ileum of the NCUG group in comparison with the NBW group (Fig. 4; *P* < 0.05). Meantime, higher mRNA expression levels of *NDUF A1*, *NDUF A6, NDUF A13*, *NDUF B1* in the jejunum, *NDUF A13* in the ileum of CUG piglets than the NBW piglets were also observed (*P* < 0.05). The mRNA expression levels of duodenal and jejunal *NDUF A1*, *NDUF A13*, *NDUF B1*, ileal *NDUF A1*, *NDUF A6*, *NDUF A13*, *NDUF B1* were higher in CUG compared with the NCUG group (*P* < 0.05). Additionally, compared to NBW group, CUG group has higher protein expression of P-AMPK/AMPK in the jejunum and ileum (*P* < 0.05). The protein expression of P-AMPK/AMPK was upregulated in the duodenum, jejunum, and ileum of the CUG piglets compared to the NCUG piglets (*P* < 0.05).

### Intestinal redox status

**Fig. 5:**
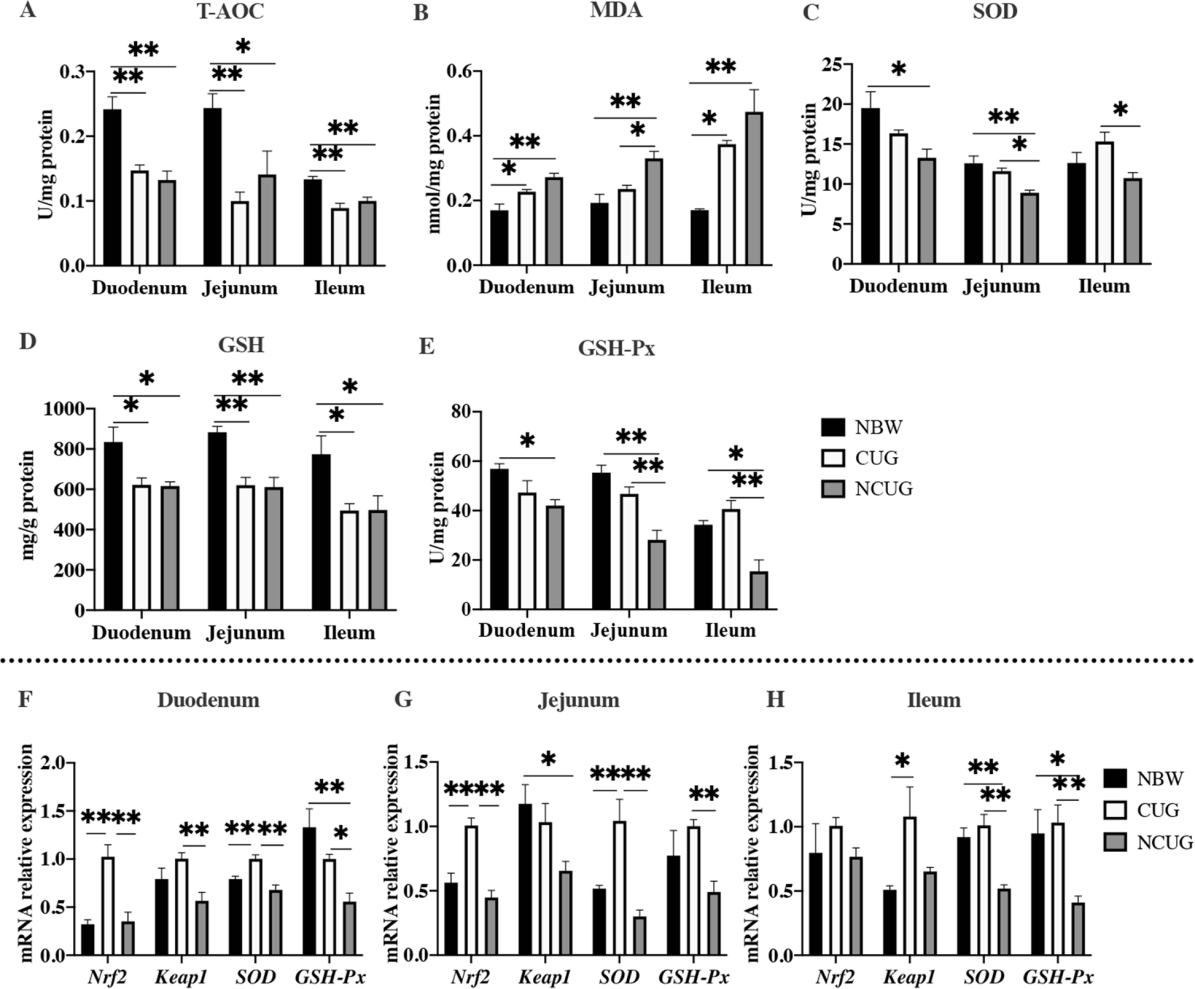
Intestinal antioxidant status and the mRNA expression of Nrf2 pathway in NBW, CUG and NCUG piglets on 26 d. **A**–**E** The activities of T-AOC, MDA, SOD, GSH and GSH-Px of duodenum, jejunum and ileum.**F** The mRNA expression levels of *Nrf2*, *Keap1*, *SOD*, *GSH-Px* in the duodenum. **G** The mRNA expression levels of *Nrf2*, *Keap1*, *SOD*, *GSH-Px* in the jejunum. **H** The mRNA expression levels of *Nrf2*, *Keap1*, *SOD*, *GSH-Px* in the ileum. Dates are presented as means ± SEM (*n* = 4). NBW, normal birth weight. CUG, catch-up growth. NCUG, not catch-up growth. *P < 0.05; **P < 0.01

The results obtained on the status of redox in the intestinal mucosa are presented in Fig. 5. Compared to the NBW group, T-AOC and GSH activity were decreased in the duodenum, jejunum and ileum of both CUG and NCUG piglets. Meantime, in contrast with NBW piglets, MDA concentration was increased in the duodenum and ileum of CUG piglets, MDA concentration was also increased in the duodenum, jejunum and ileum of NCUG piglets, SOD activity was decreased in the duodenum and jejunum of NCUG piglets, GSH-Px activity was decreased in the duodenum, jejunum and ileum of NCUG piglets (*P* < 0.05). SOD and GSH-Px in CUG group had no difference with NBW group (*P* > 0.05). Nevertheless, compared to the NCUG group, the SOD activity and GSH-Px content were higher in the jejunum and ileum of CUG group (*P* < 0.05). MDA of jejunum in CUG group was lower in comparison with the NCUG group (*P* < 0.05).

To further reveal the molecular mechanism of CUG in regulating antioxidant capacity in the intestinal mucosa, mRNA expressions of Nrf2 pathway (*Nrf2*, *Keap1*, *SOD* and *GSH-Px*) were determined, as shown in Fig. 5F, G, H. Compared to the NBW group, NCUG showed lower mRNA expression of duodenal *GSH-Px*, jejunal *Keap1*, ileal *SOD* and *GSH-Px*, CUG showed higher mRNA expression of duodenal and jejunal *Nrf2* and *SOD,* ileal *Keap1* (*P* < 0.05). We observed a higher mRNA expression of duodenal *Nrf2*, *Keap1*, *SOD*, *GSH-Px*, jejunal *Nrf2, SOD*, *GSH-Px*, ileal *SOD* and *GSH-Px* in CUG compared with the NCUG (*P* < 0.05).

### Intestinal inflammation status

**Fig. 6:**
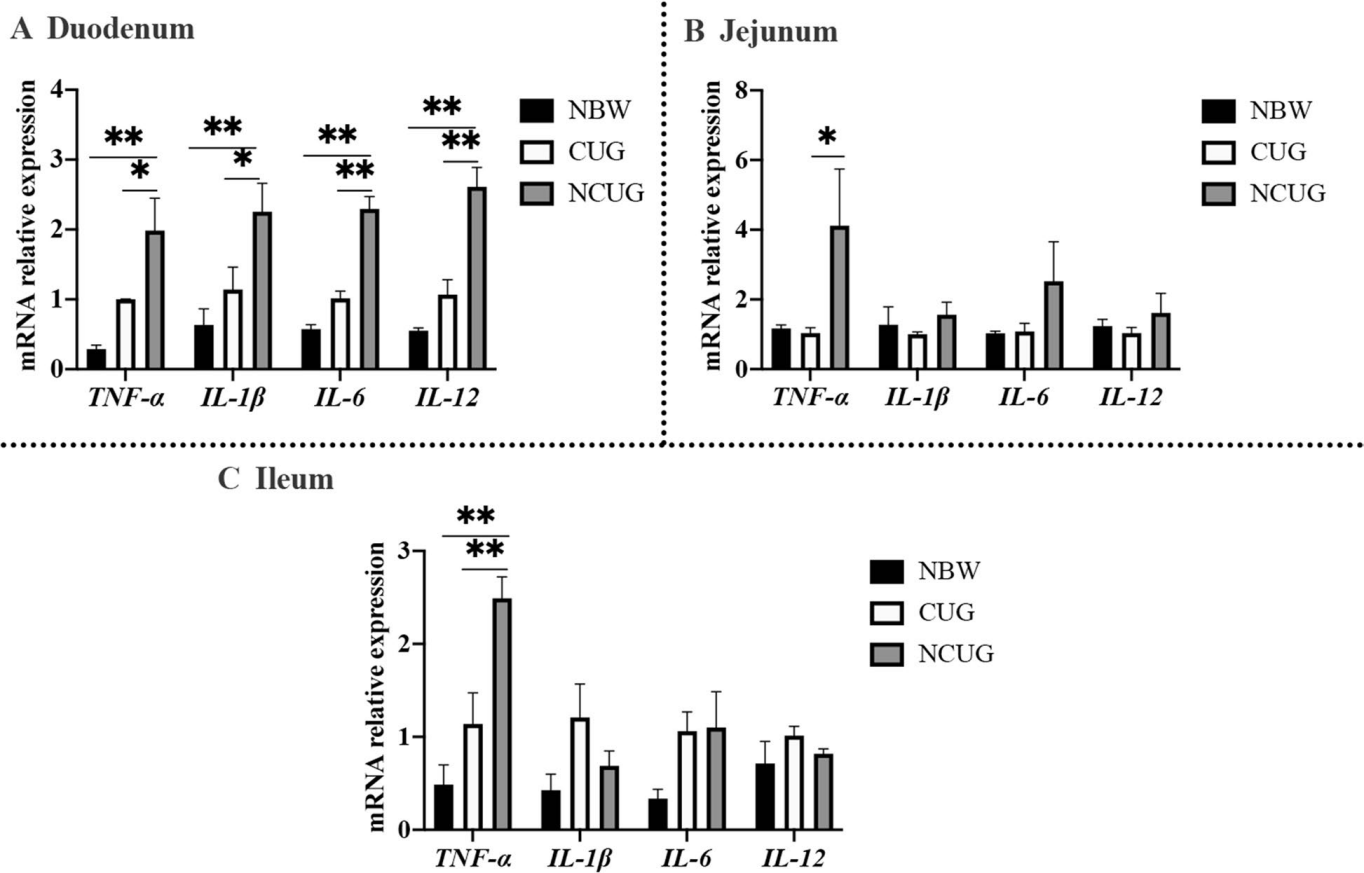
The mRNA expression of intestinal inflammation status of NBW, CUG and NCUG piglets on 26 d. **A** The mRNA expression levels of *TNF-α*, *IL-1β*, *IL-6* and *IL-12* in the duodenum. **B** The mRNA expression levels of *TNF-α*, *IL-1β*, *IL-6* and *IL-12* in the jejunum. **C** The mRNA expression levels of *TNF-α*, *IL-1β*, *IL-6* and *IL-12* in the ileum. Dates are presented as means ± SEM (*n* = 4). NBW, normal birth weight. CUG, catch-up growth. NCUG, not catch-up growth. *P < 0.05; **P < 0.01

**Fig. 7:**
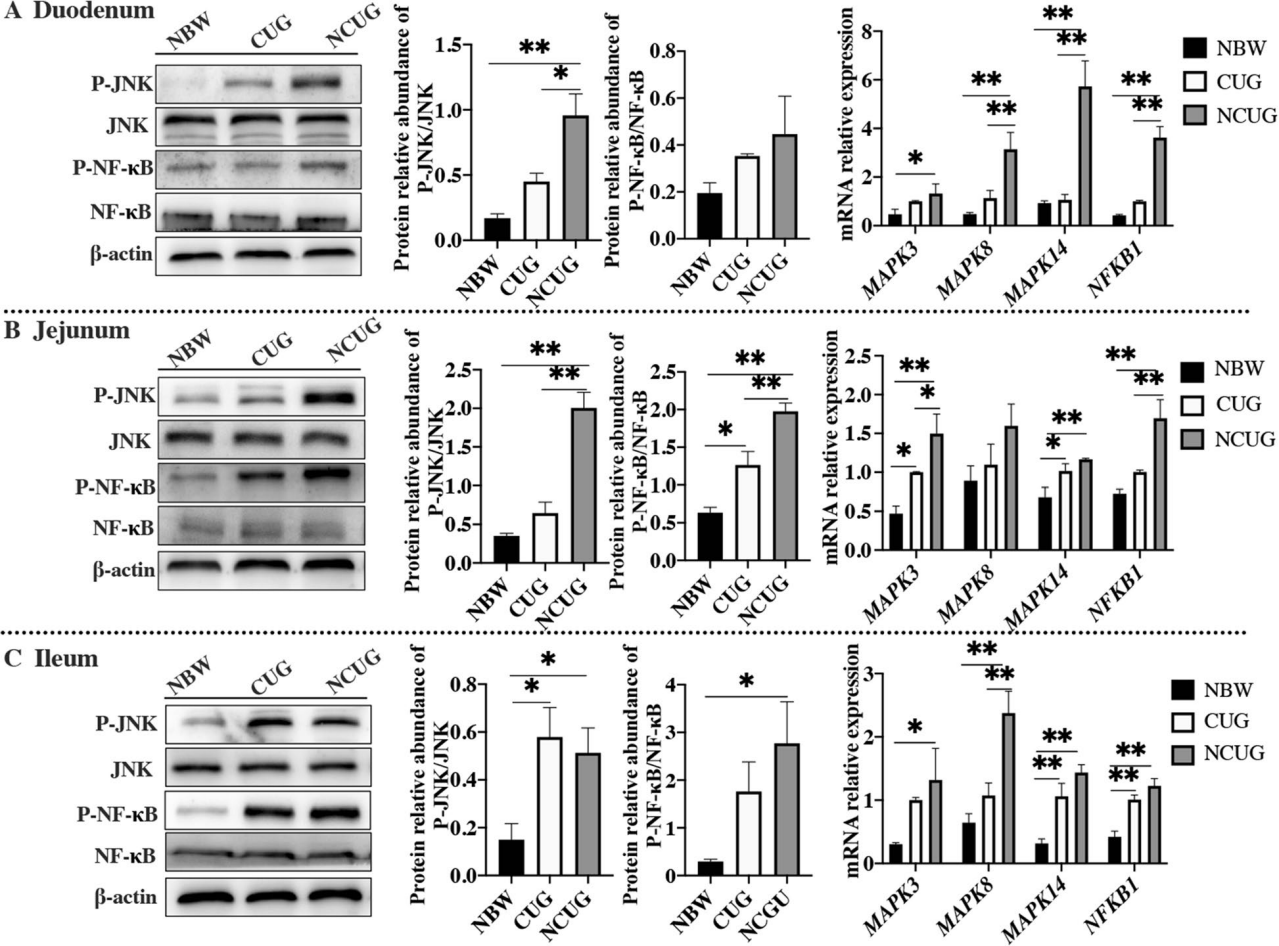
The intestinal protein and mRNA expression of NF-κB and MAPK signaling pathways of NBW, CUG and NCUG piglets on 26 d. **A** Protein expression of P-JNK/JNK and P-NF-κB/NF-κB, mRNA expression of *MAPK3*, *MAPK8*, *MAPK14* and *NFKB1* in the duodenum. **B** Protein expression of P-JNK/JNK and P-NF-κB/NF-κB, mRNA expression of *MAPK3*, *MAPK8*, *MAPK14* and *NFKB1* in the jejunum. **C** Protein expression of P-JNK/JNK and P-NF-κB/NF-κB, mRNA expression of *MAPK3*, *MAPK8*, *MAPK14* and *NFKB1* in the ileum. Dates are presented as means ± SEM (*n* = 3 for protein expression; *n* = 4 for mRNA expression). NBW, normal birth weight. CUG, catch-up growth. NCUG, not catch-up growth. *P < 0.05; **P < 0.01

As shown in Fig. 6, NCUG groups exhibited significantly increased the mRNA expression of *TNF-α*, *IL-1β*, *IL-6* and *IL-12* in duodenum, *TNF-α* in ileum than the NBW group (*P* < 0.05). There were no differences in *TNF-α*, *IL-1β*, *IL-6* and *IL-12* in duodenum, jejunum and ileum between CUG and NBW piglets (*P* > 0.05). Compared to NCUG piglets, CUG indicated lower mRNA expression of duodenal *TNF-α*, *IL-1β*, *IL-6* and *IL-12*, jejunal and ileal *TNF-α* (*P* < 0.05). In addition, western blot analysis revealed that the NCUG piglets showed higher ratio of duodenal P-JNK/JNK, jejunal and ileal P-JNK/JNK and P-NF-κB/NF-κB compared with NBW piglets (Fig. 7; *P* < 0.05). Also, CUG piglets showed higher ratio of jejunal P-NF-κB/NF-κB and ileal P-JNK/JNK compared with NBW piglets (*P* < 0.05). Meanwhile, the CUG piglets showed lower protein levels of duodenal P-JNK/JNK, jejunal P-JNK/JNK and P-NF-κB/NF-κB compared with the NCUG piglets (*P* < 0.05). Similarly, in contrast to NBW piglets, the mRNA expression of duodenal and ileal *MAPK3*, *MAPK8*, *MAPK14* and *NFKB1*, jejunal *MAPK3* and *NFKB1* were upregulated in NCUG, while the mRNA expression of jejunal *MAPK3* and *MAPK14*, ileal *MAPK14* and *NFKB1* were upregulated in CUG (Fig. 7; *P* < 0.05). Moreover, CUG showed markedly downregulated mRNA levels of *MAPK8*, *MAPK14,* and *NFKB1* in the duodenum, *MAPK3* and *NFKB1* in the jejunum, and *MAPK8* in the ileum than that in the NCUG group (*P* < 0.05).

### Intestinal tight junction proteins levels

**Fig. 8:**
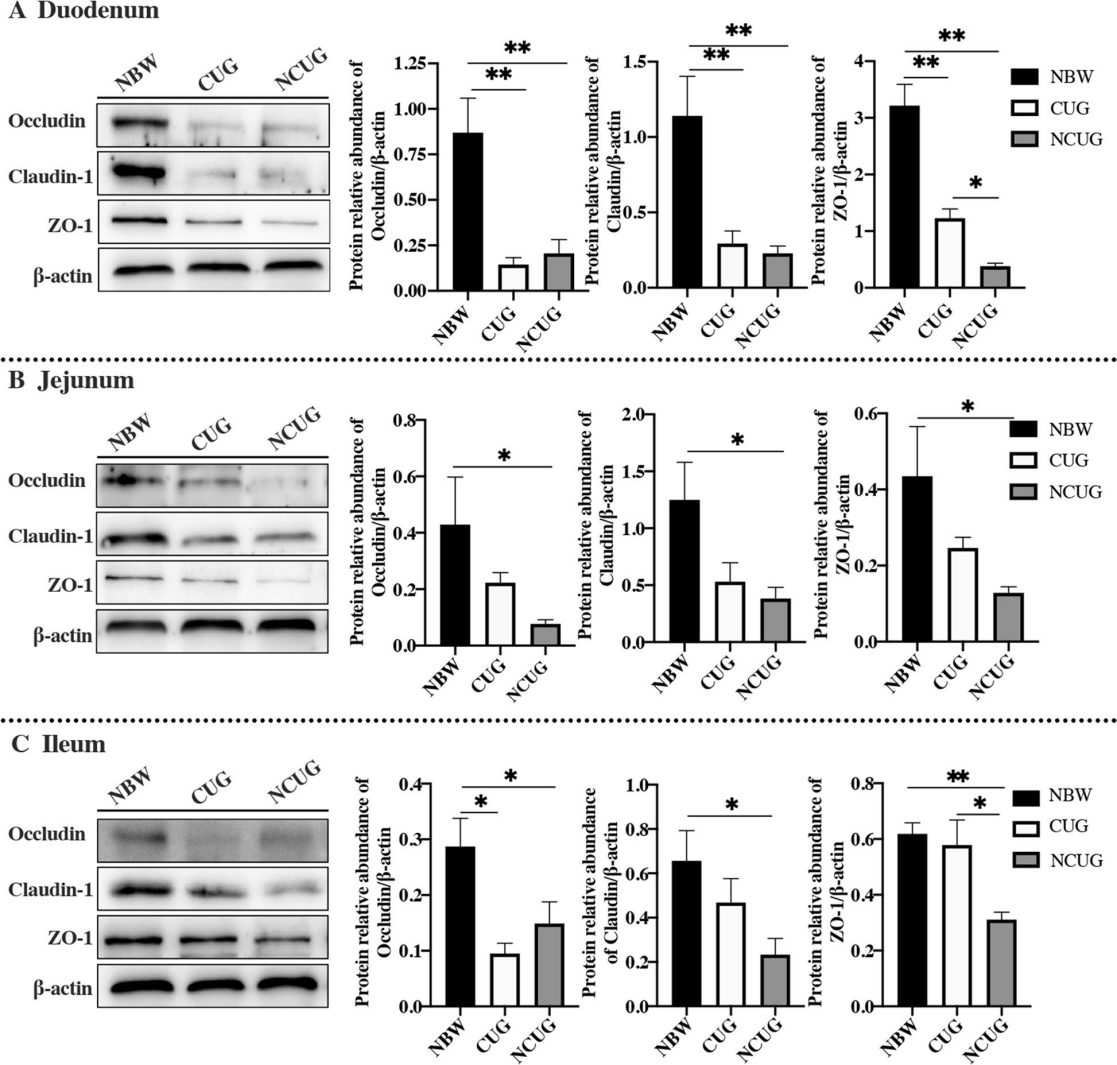
Intestinal tight junction proteins levels of NBW, CUG and NCUG piglets on 26 d. **A** The protein expression of Occludin, Claudin-1 and ZO-1 in the duodenum. (**B**) The protein expression of Occludin, Claudin-1 and ZO-1 in the jejunum. (**C**) The protein expression of Occludin, Claudin-1 and ZO-1 in the ileum. Dates are presented as means ± SEM (*n* = 3). NBW, normal birth weight. CUG, catch-up growth. NCUG, not catch-up growth. *P < 0.05; **P < 0.01

Western blot analysis showed that the protein levels of duodenal, jejunal and ileal Occludin, Claudin-1, and ZO-1 were decreased in NCUG piglets compared with the NBW piglets (Fig. 8; *P* < 0.05). CUG piglets decreased the protein levels of duodenal Occludin, Claudin-1, ZO-1, ileal Occludin compared with the NBW piglets (*P* < 0.05). Further, in contrast to NCUG piglets, no significant CUG action for Occludin, Claudin-1 was observed in the duodenum and jejunum (*P* > 0.05), but evident higher protein expression levels of ZO-1 were observed in the duodenum and ileum of CUG piglets (*P* < 0.05).

### Microbiota populations

**Fig. 9:**
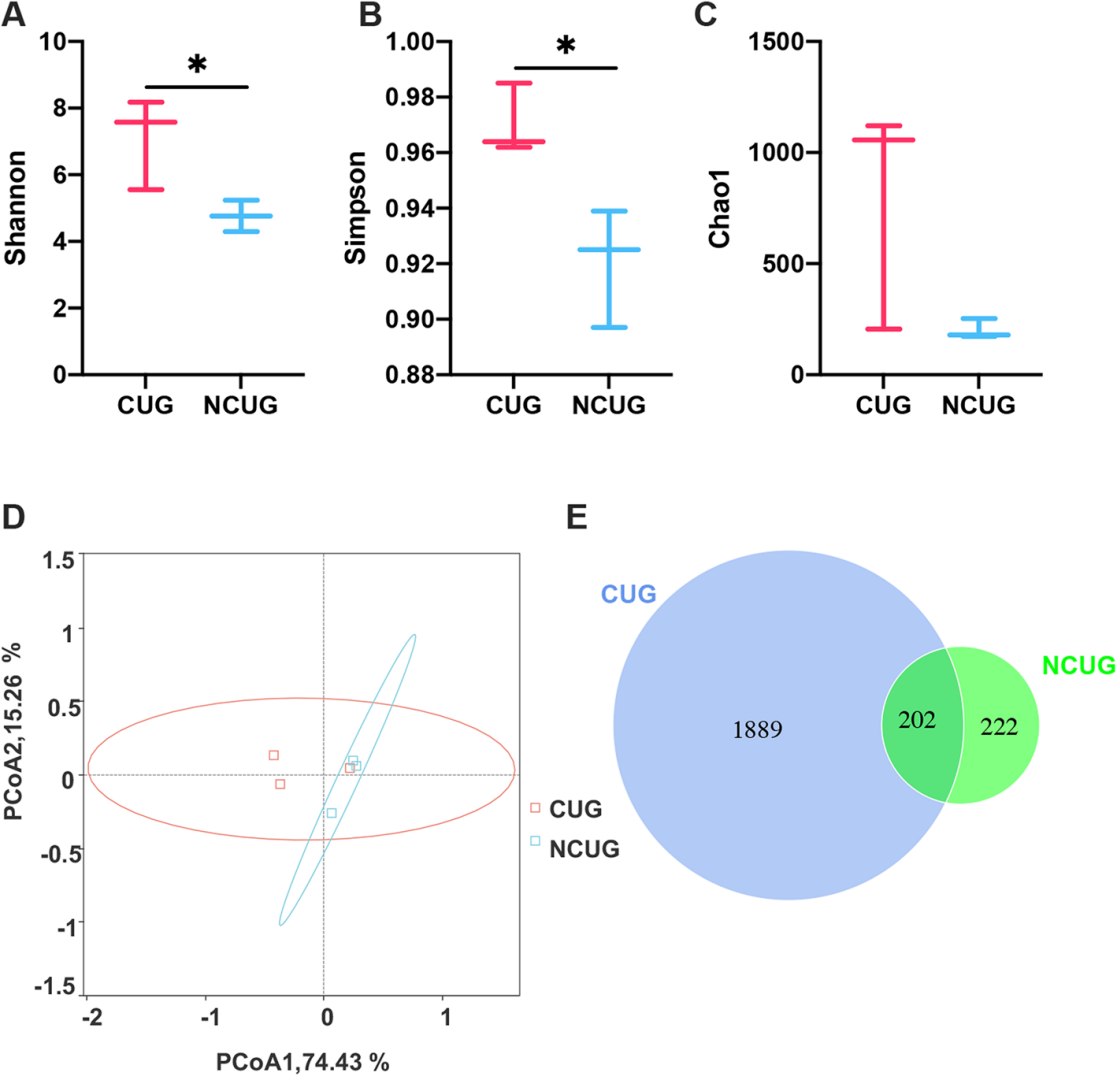
The shifts in feces alpha and beta diversity of CUG and NCUG piglets on 7 d. **A** Shannon index of microbiota. **B** Simpson index of microbiota. **C** Chao1 index of microbiota. **D** Principal component analysis (PCoA) scores plot. **E** Venn diagram for the OTUs. CUG, catch-up growth; NCUG, not catch-up growth. *P < 0.05

16S rRNA gene sequencing technology was used to compare the feces bacterial community composition of different pattern CUG piglets and NCUG piglets. After sequencing, a total of 2091 OTUs were identified from CUG and 424 OTUs were identified from NCUG, 202 shared OTUs out of the total OTUs overlapped between the two groups (Fig. 9E). In this study, the alpha diversity of the feces microbiota expressed by Shannon, Simpson and Chao1 (Fig. 9A, B, C). Compared with the NCUG group, Shannon and Simpson were significantly higher in the CUG group (*P* < 0.05), while there were no significant differences in Chao1 (*P* > 0.05). For beta diversity, the PCoA analyses based on weighted_unifrac distance showed that the microbiota there was no obvious tendency to separate CUG from NCUG (Fig. 9D).

**Fig. 10:**
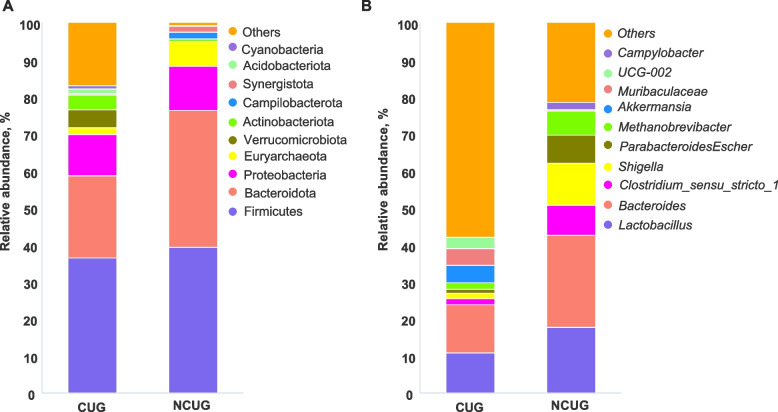
The relative abundance of CUG and NCUG feces microbiota community at the phylum and genus level on 7 d. **A** Relative abundance at the phylum level. **B** Relative abundance at the genus level. CUG, catch-up growth; NCUG, not catch-up growth

In order to further determine the changes in fecal microbiota composition, the dominant phylum and genus of each group were analyzed. At the phylum level (Fig. 10A), Firmicutes, Bacteroidetes, Proteobacteria and Euryarchaeota predominantly mainly constituted the fecal microbiota of piglets. Compared with the NCUG group, the relative abundance of Firmicutes in the CUG group decreased from 39.30% to 36.4%, and the relative abundance of Bacteroidetes, Proteobacteria and Euryarchaeota decreased from 36.9%, 11.9%, 6.57% to 22.3%, 11.1% and 1.81%, respectively. The ratio of Firmicutes/Bacteroidetes was increased from 1.06% to 1.64% in CUG compared with the NCUG group. At the genus level, a total of 337 bacterial genera were annotated, among which the top 10 were shown in Fig. 10B.

Compared with the NCUG group, the relative abundance of *Lactobacillus*, *Bacteroides*, *Clostridium_sensu_stricto_1* and *Escherichia_Shigella* decreased from 17.65%, 24.87%, 8.11%, 11.49% to 10.91%, 12.96%, 1.65% and 1.41%, respectively.

**Fig. 11:**
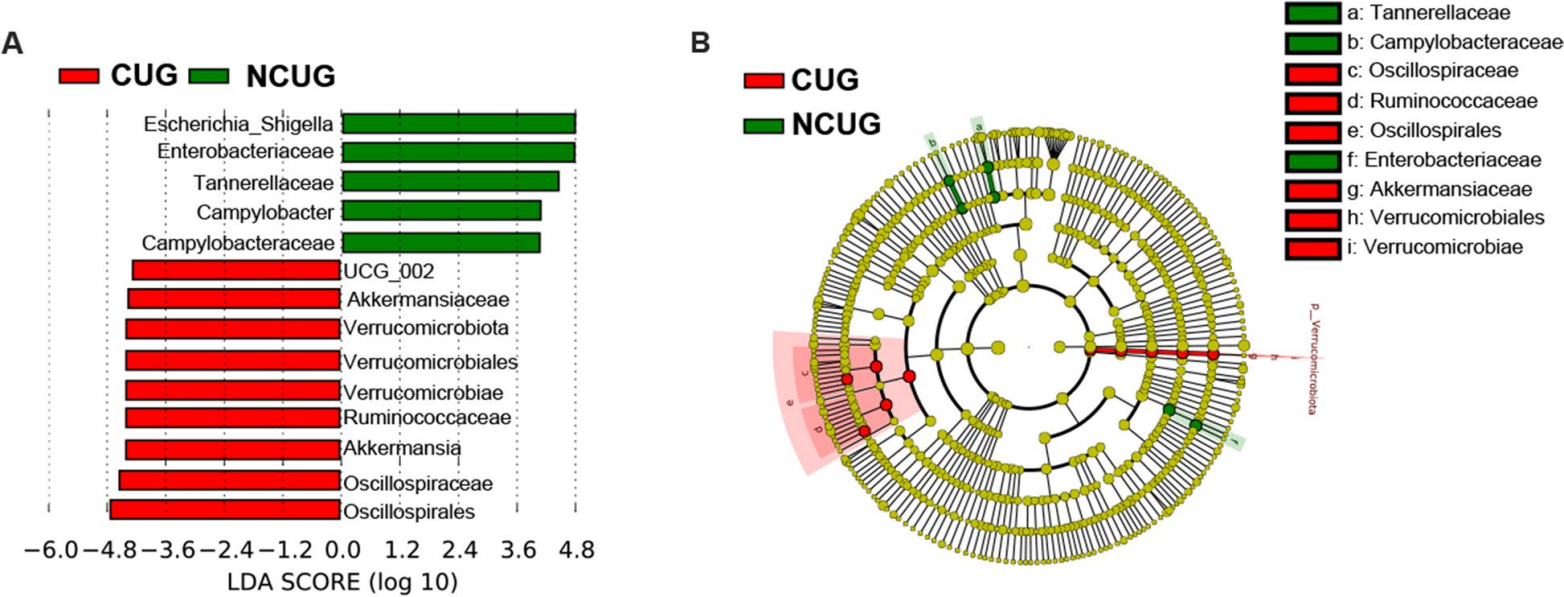
Linear discriminant analysis combined effect size (LEfSe) measurement analysis of microbiota in the feces contents of CUG and NCUG piglets on 7 d. **A** Linear discriminant analysis (LDA) score from phylum to genus level of the fecal microbiota, and the score ≥ 2 means significant. **B** Cladogram of LEfSe shows taxonomic profiling from the phylum to genus level, the yellow node represents no difference, but other color nodes represent significant difference

The effect of microbial abundance of each species on the differential effect was evaluated by LDA (LDA threshold > 3.6). The results (Fig. 11A, B) indicated that the fecal microbiota composition was affected by CUG. A higher richness of *UCG_002*, *gut_metagenome*, Akkermansiaceae, Verrucomicrobiota, Verrucomicrobiales, Verrucomicrobiae, Ruminococcaceae, *Akkermansia*, Oscillospiraceae and Oscillospriales in CUG group as well as *Escherichia_Shigella*, Enterobacteriaceae, Tannerellaceae, *Parabacteroides*, *Campylobacter* and Campylobacteraceae in NCUG group were observed in the feces of piglets.

**Fig. 12:**
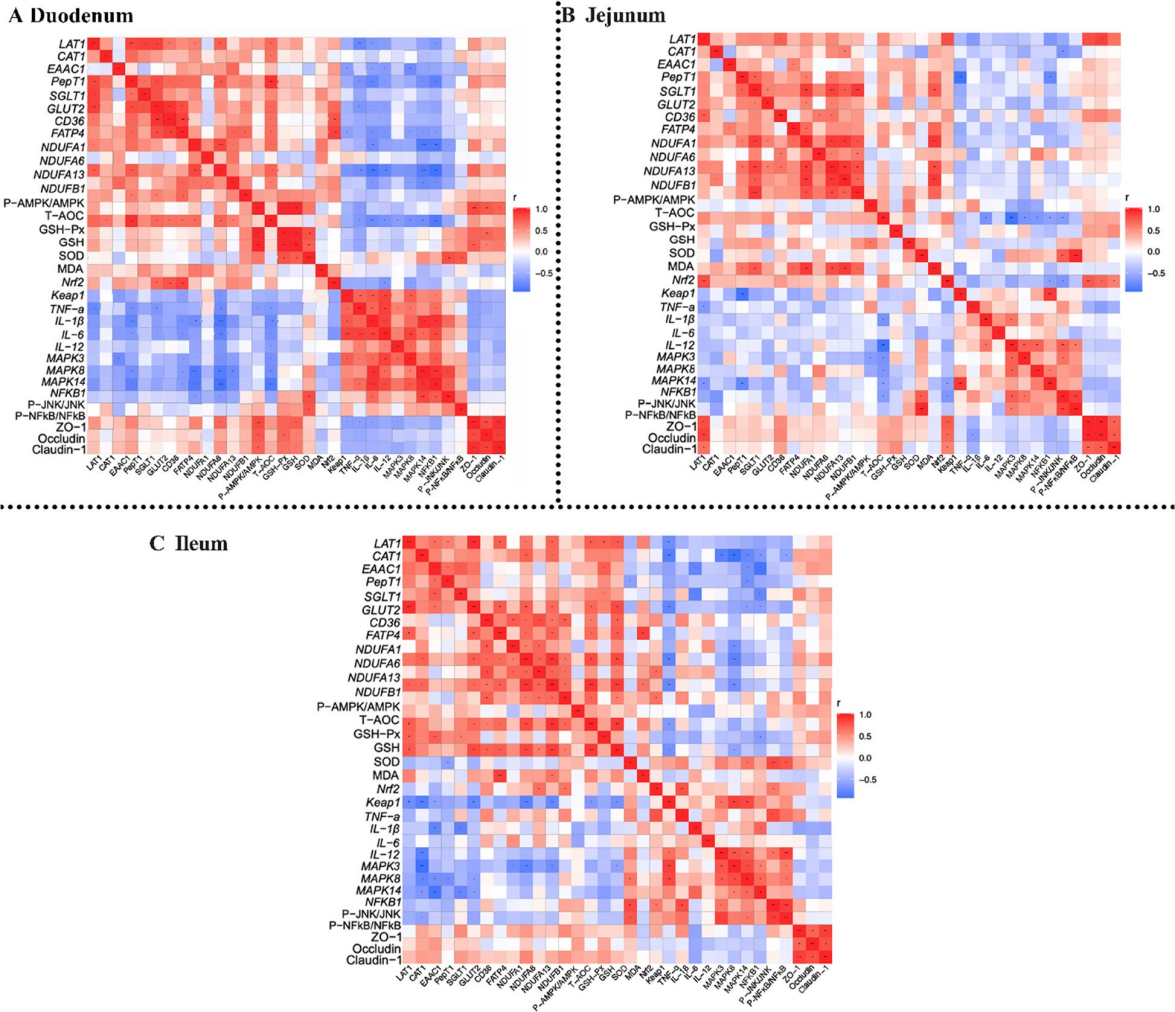
Heatmap of Pearson’s correlation coefficients between intestinal nutrition transport, energy metabolism, antioxidant status, immune status, inflammatory pathways and intestinal barrier function. **A** Heat map of duodenal correlation analysis. **B** Heat map of jejunal correlation analysis. **C** Heat map of ileal correlation analysis. In the panel, red with a *P* < 0.05 represents a significant positive correlation, blue with a *P* < 0.05 represents a significant negative correlation, and white represents no correlation. *P < 0.05; **P < 0.01

### Correlation analysis

A series of correlation analyses between gut nutrient transport, energy metabolism, antioxidant status, inflammatory responses, and gut permeability in piglets was revealed (Fig. 12). In duodenum, nutrient transport was positively correlated with *NDUF A1*, *NDUF A13*, *NDUF B1*, P-AMPK/AMPK and GSH-Px, and negatively correlated with *TNF-α*, *IL-1β*, *IL-6*, *IL-12*, *MAPK8*, *MAPK14* and *NFKB1*. Energy metabolism was positively correlated with GSH-Px, and negatively correlated with *TNF-α*, *IL-1β*, *IL-6*, *IL-12*, *MAPK3*, *MAPK8*, *MAPK14*, *NFKB1*. Antioxidant status was positively correlated with ZO-1, Occludin and Claudin, and negatively correlated with *IL-1β*, *IL-6*, *IL-12*, *MAPK3*, *MAPK8*, *MAPK14*, *NFKB1*. Inflammatory responses were negatively correlated with ZO-1. In jejunum, that nutrient transport was positively correlated with *NDUF A1*, *NDUF A13,* NDUF B1, P-AMPK/AMPK, *Nrf2*, ZO-1, Occludin and Claudin-1, and negatively correlated with *TNF-α*, *IL-1β*, *NFKB1*. Energy metabolism was positively correlated with *Nrf2*. Antioxidant status was negatively correlated with *IL-6*, *MAPK3*, *MAPK8*, *MAPK14*, *NFKB1* and P-JNK/JNK. In ileum, that nutrient transport was positively correlated with *NDUF A1*, *NDUF A6*, *NDUF A13*, *NDUF B1*, P-AMPK/AMPK, GSH-Px, GSH, SOD, and negatively correlated with *TNF-α*, *IL-6*, *MAPK3*, *MAPK8*, *MAPK14* and *NFKB1.* Energy metabolism was positively correlated with, GSH-Px, SOD, *Nrf2*, *Keap1*, and negatively correlated with *TNF-α* and *MAPK8*.

## Discussion

It is well known that IUGR has a permanent stunting effect on the postnatal growth of piglets due to accompanied impaired development of momentous organs, which potentially further deteriorate pre-existing impaired growth [24]. The gut plays a vital role in postnatal development of piglets and its abnormal development usually causes feeding intolerance, nutrient absorption problems and necrotizing enterocolitis [25–27], which negatively influence nutrients obtainment and lead to a poorer growth rate of IUGR piglets. Previous studies have shown that both newborn and weaned piglets with IUGR were characterized by damaged intestinal structure, such as decreased VH, increased CD and decreased VH/CD ratio [10, 25, 28]. In our present study, it is not surprising to find the destroyed intestinal morphology in NCUG weanling piglets similar to the previous study, but it is interesting that CUG piglets had much better intestinal structure, almost as good as NBW piglets. Our result indicated that the intestinal development from intrauterine in CUG has been restored during the suckling period, which means the potential for improved ability to get necessary nutrients for growth.

The nutrients uptake is mainly facilitated by their corresponding transporters including amino acids transporters, glucose transporters, and fatty acids transporters distributed aligned on the intestinal epithelium [29]. Nutrient uptake is not only the first step for nutrient absorption, but also the main source of energy and nutrients for intestinal metabolism and development. Accumulating evidence showed that intestinal growth stagnation and dysfunction in piglets with IUGR are accompanied by a series of nutrient transporter changes and disorders of nutrient and energy metabolism [30–32]. It has been reported that mRNA expression of several nutrient transporters including *SLC1A1*, *SLC7A7*, *SLC7A9*, *PepT1*, *FABP4, SLC5A1*, and *GLUT2* was significantly reduced in the jejunum of IUGR piglets [30], implying that inadequate nutrient uptake is strongly associated with impaired gut development and function. Glucose acts as the primary ATP producer and provides the most energy to intestinal epithelial cells to perform nutrient transportation. In current study, we found that the mRNA expression of *GLUT2* and *SGLT1*, the two major glucose transporters in intestine were upregulated in CUG piglets compared with NCUG piglets, while significantly higher mRNA expression of jejunal *SGLT1* in CUG piglets compared with the other two groups. The better uptake capacity of glucose in small intestine of CUG piglets could provide sufficient energy to support intestinal development and ATP dependent nutrient transport, which is the foundation for intestinal function. We also found that the mRNA expression of amino acid transporters, including *LAT1*, *CAT1* and *PepT1* were significantly higher in CUG piglets than those of NCUG. This observation implied promoted amino acid obtaining capacity in CUG piglets, which might be a reason for their higher growth rate during lactation since amino acids are precursors of protein synthesis [13]. In addition, the mRNA expression of fatty acid transporters *FATP4* and *CD36* was also consistently found higher in CUG intestine in this study. Collectively, the higher mRNA expression of nutrient transporters in the intestine of CUG piglets suggested improved nutrients uptake capability potentially providing more substance for accelerated growth.

Intestinal proliferation, renewal, and active transport of nutrients entirely depend on ATP produced by mitochondrial oxidative phosphorylation [33–35]. Mitochondrial dysfunction has been shown to limit energy production resulting in malabsorption and intestinal disorders in IUGR piglets [20, 33, 35, 36]. Mitochondrial complex I is the first and the most important rate-limiting step in the mitochondrial ETC, providing a major proton-motive force that drives ATP synthesis, and its activity is positively correlated with ATP production [37]. A previous study showed complex I of ETC and ATP synthase activities were both decreased in intestine of IUGR piglets [38]. In present study, mRNA expression of ETC complex I was also shown to decrease in NCUG, while no obvious difference between CUG and NBW piglets, suggesting that the characterized energy lack in IUGR intestine has been repaired in CUG, which is also consistent with the enhanced nutrients transporters. AMPK has been widely reported as a critical sensor of cell energy status regulating cell metabolism to maintain energy homeostasis [39]. It is well known that AMPK activation could directly promote ATP production by increasing the activity or expression of proteins involved in catabolism, while switching off biosynthetic pathways to meet cellular energy requirement [30, 40]. Zhang et al. [41] reported that the AMPK signaling activation was inhibited in intestine of weaning IUGR piglets resulting in abnormal energy status and reduced ATP production. In current study, we did not find significant differences in the ratio of P-AMPK/AMPK protein expression between NCUG and NBW piglets’ intestines but observed significant upregulation of jejunum and ileum in the CUG. Hence, these results suggested that activation of intestinal AMPK and ETC complex I in CUG piglets repair intestinal energy metabolism and ensure the supply of ATP required for intestinal nutrient transport, thus promoting the absorption of intestinal nutrients and leading to body recovery in CUG piglets.

Redox imbalance in young animals causes uncontrolled oxidative stress with local and systemic damage, thus reducing the growth performance and increasing the risk of metabolic syndrome in adulthood [42]. Previous studies have observed a deficiency of antioxidative system in IUGR piglets compared with their normal counterparts [43–45]. It has also been pointed out that extensive oxidative stress might be the vital factor leading to intestinal injury in IUGR piglets [45]. In this case, we compared the redox controlling ability among the intestine from three groups of piglets and a significantly higher GSH-Px and SOD content was observed in both the jejunum and ileum in CUG piglets than those NCUG piglets. Meanwhile, the activity of MDA was lower in jejunum of CUG piglets than NCUG piglets. These results illustrate that intestine of CUG piglets has a recovered antioxidant capacity and less oxidative damage. Nrf2 plays an important role in the antioxidant response and inflammation. Under normal conditions, Nrf2 and Keap1 bind stably in the cytoplasm. However, under oxidative stress, Nrf2 is isolated from Keap1 and translocated to the nucleus, where it activates antioxidant gene targets [46]. We found the expressions of several genes involved in Nrf2 signaling pathway such as *Nrf2*, *Keap1*, *SOD* and *GSH-Px* in the duodenum, jejunum and ileum were significantly higher in CUG intestine than those of NCUG piglets, which may offer a further explanation for the improved antioxidative ability of CUG.

Increased oxidative stress in intestinal tissue can trigger a series of inflammatory responses leading to the overexpression of inflammatory cytokines and eventually severe inflammatory bowel disease [47, 48]. A growing number of studies have shown that oxidative stress activated the NF-κB signal with other related transcription factors to stimulate rapid secretion and accumulation of IL-6, IL-1β, and TNF-α [49]. IUGR piglets have been reported prone to intestinal inflammatory diseases and this situation often involves an increase in pro-inflammatory cytokines and a decrease in anti-inflammatory cytokines [50]. Our dates found that the mRNA abundances of *TNF-α*, *IL-1β*, *IL-6* and *IL-12* were significantly downregulated of CUG group compared with NCUG group. The MAPK subfamily, concluding three major subfamilies, P38, JNK, and ERK, mediate inflammatory induced signal transduction pathways [51]. The NF-κB pathway regulates genes involved in immune and inflammatory processes and its activation could accelerate the release of pro-inflammatory cytokines leading to tissue damage ultimately [52]. In the present study, we observed the expression of MAPK and NF-κB significantly downregulated in CUG piglets compared with NCUG piglets, which further indicates that CUG piglets have lower levels of intestinal inflammation. The decrease of intestinal inflammation in CUG piglets may be due to the higher antioxidant capacity of the intestine, which improves the antiviral ability of the body, thus contributing to the healthy growth of CUG piglets.

The overexpression of pro-inflammatory cytokines could lead to intestinal epithelial cell membrane damage and disruption of intestinal tight junctions, thereby disrupting intestinal permeability [53]. Tight junction structures are momentous parts of the intestinal epithelial barrier system [54]. The integrity of the epithelium will be damaged when the tight junctions are destroyed, leading to entering of pathogens or toxins into the systemic circulation [55]. Evidence has revealed that IUGR piglets have reduced intestinal tight junction protein expression, and intestinal permeability was nearly twice as high as that of NBW piglets, damaging the intestinal physical barrier [43, 56]. Notably, our results showed the decreased protein expression of Occludin, Claudin-1 and ZO-1 in CUG and NCUG piglets. ZO-1 has been upregulated in CUG piglets compared to NCUG piglets, especially in duodenum and ileum, with no effects of treatments on Occludin and Claudin-1. Occludin and Claudin-1 are the main cytoplasmic transmembrane proteins, while ZO-1 is the most important cytoplasmic adaptor protein [55]. Our findings suggest a recovered physical intestinal barrier in CUG piglets around weaning, which can help protect the body from external pathogenic bacteria and toxins and enhance the immune capacity of the body, thus achieving the function of regulating the intestinal health of the body and ensuring the normal growth of the body.

Intestinal microbiota plays an important role in regulating host animal’s immune and physiological functions and numerous researches have shown a high correlation between intestinal microbiota and intestinal barrier function [57]. It has been reported that IUGR alters the intestinal microbiome, with significantly higher levels of Gram-negative bacteria that cause systemic inflammation [58]. Our results showed that microbial Simpson and Shannon indices in alpha diversity were higher in the CUG group. Otherwise, higher alpha diversity is thought to be beneficial for maintaining host intestinal homeostasis. Further analysis by beta diversity of microbiota, the microbial composition in feces of piglets showed that there was no significant difference between CUG and NCUG piglets at 7 d. However, our LEfSe analysis showed that the CUG group had a higher population of the Ruminococcaceae and *Ruminococcaceae_UCG_002*. Previous studies have shown that the presence of Ruminococcaceae is associated with the maintenance of gut health and the presence of numerous carbohydrate-active enzymes [59]. In addition, the LEfSe proved CUG piglets had a markedly higher relative abundance of the Verrucomicrobiota, Verrucomicrobiales, Verrucomicrobiae, Akkermansiaceae and *Akkermansia* than NCUG groups. Verrucomicrobiota is regarded as potentially beneficial bacterium in the gut, and in previous studies it was found to be more abundant in the homeostasis of the gut [60, 61]. Akkermansiaceae is the only known species of the phylum Verrucomicrobiota in mammals [62], which can improve intestinal health by adhering to intestinal epithelial cells and enhancing monolayer integrity of intestinal epithelial cells [63]. Higher Oscillospirales and Oscillospiraceae levels were found in CUG piglets, previous research showed that the Oscillospiraceae can produce butyrate and inhibit the growth of pathogenic bacteria in gastrointestinal tract [64]. The Enterobacteriaceae showed a higher population in NCUG piglets in the LEfSe analysis, which was believed to play an important role in developing piglet diarrhea and seriously affect the barrier function of the animal intestinal tract [65]. Meantime, in contrast to CUG piglets, the Campylobacteraceae, *Campylobacter* and Tannerellaceae were higher in NCUG piglets, which were one of the main causes of diarrhea and can cause acute gastroenteritis [66, 67]. Taken together, these changes suggest that stability of intestinal ecosystem in CUG piglets may be attributed to the higher healthy beneficial bacteria and lower underlying pathogenic microbiota.

## Conclusion

In summary, compared with NCUG piglets, intestinal nutrient transport, energy metabolism, antioxidant capacity and intestinal barrier function are better recovered in CUG piglets, while intestinal inflammation and harmful microbiota were mitigated in CUG piglets. This study is the first to investigate the changes in intestinal development function of CUG piglets during weaning and the results also could provide a detailed understanding of intestinal development in infants with CUG due to the limitation of samples obtaining in humans studies.

## Data Availability

The data analyzed during the current study are available from the corresponding author on reasonable request.

## Abbreviations

ANOVA: Analysis of variance
CUG: Catch-up growth
CD: Crypt depth
ETC: Electron transport chain
GSH-PX: Glutathione peroxidase
GSH: Reduced glutathione
NBW: Normal birth weight
Nrf2: Nuclear factor erythroid 2-related factor
T-AOC: Total antioxidant capacity
IUGR: Intrauterine growth restriction
LDA: Linear discriminant analysis
LEfSe: The linear discriminant analysis effect size
MDA: Malonaldehyde
PCoA: Principal coordinate analysis
PVDF: Polyvinylidene difluoride
SOD: Superoxide dismutase
VH: Villi height
